# Adherens junctions remain dynamic

**DOI:** 10.1186/1741-7007-8-34

**Published:** 2010-04-08

**Authors:** Matthias M Falk

**Affiliations:** 1 Department of Biology, Lehigh University, Bethlehem, PA, USA

## Abstract

One of the four principal categories of cell-cell junctions that hold together and shape distinct tissues and organs in vertebrates, adherens junctions (AJs) form cell-cell contacts that connect transmembrane proteins with cytoskeletal actin filaments to provide architectural strength, aid in morphogenesis, and help to maintain proper tissue homeostasis. The classical organization of AJs, consisting of transmembrane cadherins and cytoplasmically attached β-catenins and α-catenins assembled together into a multiprotein complex, was once thought obligatory to craft a robust and stable connection to actin-based cytoskeletal elements, but this architecture has since been challenged and questioned to exist. In a stimulating paper published in a recent issue of *BMC Biology*, Millán *et al. *provide convincing evidence that in confluent vascular endothelial cells a novel dynamic vascular endothelial (VE)-cadherin-based AJ type exists that interacts with and physically connects prominent bundles of tension-mediating actin filaments, stress fibers, between neighboring cells. Stress fibers were known previously to link to integrin-based focal adhesion complexes but not to cell-cell adhesion mediating AJs. These new findings, together with previous results support the concept that different AJ subtypes, sharing the same transmembrane cadherin types, can assemble in various configurations to either increase barrier function and promote physical cell-cell adhesion, or to lessen cell-cell adhesion and promote cell separation and migration.

## Commentary

Cells in vertebrates including humans can be linked together by four principal types of cell-cell junctions to form tissues and organs. Each type of cell-cell junction is considered to fulfill a special function. Tight junctions (TJs) form a net-like belt of branched ridges of transmembrane proteins (claudins, occludins, tricellulin) around cells that tightly link cells together to separate apical from basolateral membrane domains, or (in the case of epithelia and vascular endothelia) to separate, outside from inside, or the lumen of blood vessels from the surrounding body, respectively. Desmosomes and adherens junctions (AJs) form patchy cell-cell contacts that connect cytoskeletal elements, such as intermediate filaments and actin filaments of neighboring cells to provide tissue strength, acting as an aid in tissue morphogenesis during development, and to maintain proper tissue organization. Gap junctions (GJs) are clusters of double-membrane-spanning hydrophilic channels made from transmembrane connexin proteins that provide direct cell-to-cell communication by allowing the passage of signaling molecules, ions, and electrical currents. All four junction types provide physical cell-cell coupling. Epithelia and endothelia, sheets of polarized single-cell layers that coat the outside and inside surface of organs such as the intestine, liver, kidneys, or the vasculature are particularly rich in cell-cell junctions and exhibit a well organized hierarchical architecture of these structures. A historical overview of the discovery of the molecular components and the intricate architecture of these various intercellular junctions has recently been published [1]. However, the more we learn, the more the known principal functions of cell-cell junctions need to be amended, and sometimes even revised.

AJs consist of a family of transmembrane cadherin proteins (epithelial (E), neuronal (N), placenta (P), vascular endothelial (VE), and so on, named after the tissue where they were first described) that interact in a homophilic, calcium-dependent manner in the extracellular space to physically link cells together. Traditionally, and as depicted in many cell biology textbooks, the cytoplasmic tails of the cadherins were thought to interact with cytoplasmic linker proteins to mediate and strengthen the stable connection between transmembrane cadherins and the actin cytoskeleton. Specifically, it has been thought that β-catenin stably binds to the carboxyterminal cytoplasmic tail of cadherins, and α-catenin stably binds to β-catenin to establish the stable connection to cortical F-actin filaments. However, this view was revised in 2005 when two complementary papers from the Nelson and Weis laboratories at Stanford University provided strong evidence that a simultaneous static interaction of all four proteins (cadherin, β-catenin and α-catenin, F-actin) does not appear to exist as previously dogmatized. Rather, the authors proposed that α-catenin represents a molecular switch that can bind either to E-cadherin/β-catenin complexes (in its monomeric conformation), or to F-actin filaments (in its homodimeric conformation), but not to both. The previous view that α-catenin would tether plasma membrane AJs and cytoplasmic actin assemblies together to establish a robust linkage between these two cellular components was not supported by their findings ([2,3] reviewed in [4,5]). Evidence presented in these papers thus suggested that the predicted linkage between cadherin-catenin complexes and cortical F-actin filaments is either non-existent, or at least remains much more dynamic than has been anticipated previously. These provocative findings, of course, spurred a surge of new investigations toward a better understanding of the mechanisms and factors that regulate the stability and dynamic remodeling of plasma membrane AJs.

**Figure 1:**
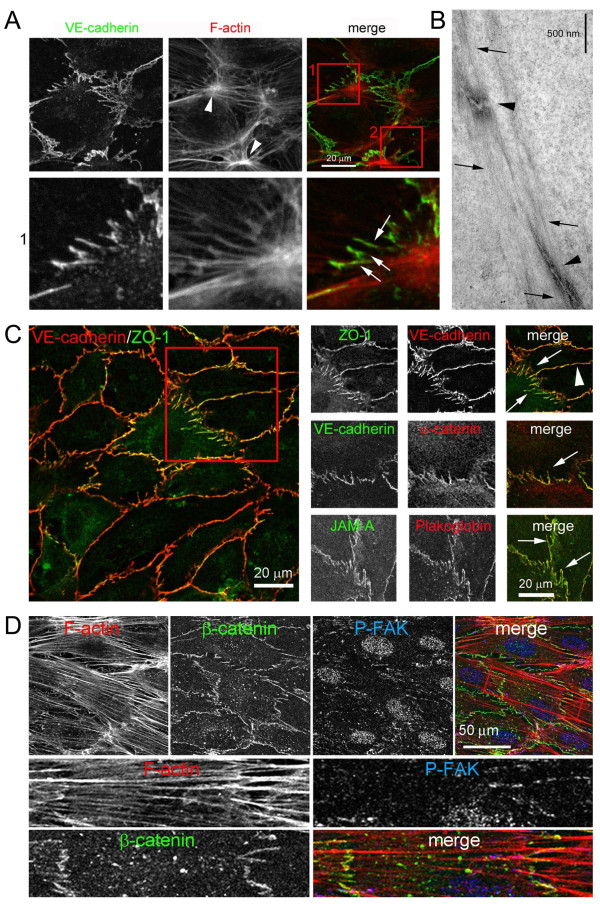
**Discontinuous adherens junctions (AJs) in confluent human umbilical vein endothelial cells (HUVECs) described by Millán *et al ***[6]. **(a-c) **Discontinuous AJs form separated clusters of vascular endothelial (VE)-cadherin-based junctions at the ends of F-actin-based stress fibers that are oriented more or less orthogonal to the adjacent cell membranes (boxed in **(c) **and marked with arrows). There they appear to connect stress fibers between adjacent cells (insert 1 in **(a)**). Discontinuous AJs contain aside from VE-cadherin, both α-catenin and β-catenin **(c, d) **and a number of additional junctional proteins such as zona occludens (ZO)-1, plakoglobin and junctional adhesion molecule A (JAM-A) **(c)**, p120-catenin, and CD99. Discontinuous AJs attached to stress fibers did not colocalize with the focal adhesion (FA) components, focal adhesion kinase (P-FAK) (d), paxillin, or talin, indicating that FAs and discontinuous AJs are distinct, separate structures. Fluorescent images **(a, c, d)**, ultrastructure **(b)**. All images are taken from Millán *et al. *[6].

In a recent issue of *BMC Biology*, Millán *et al.*, based at University College London, King's College London, UK, and Universidad Autónoma de Madrid, Spain, reported that a new type of AJ they discovered and characterized in confluent vascular endothelial cells (human umbilical vein endothelial cells (HUVECs)) and named 'discontinuous AJs', do indeed robustly interact with actin filaments [6]. Unexpectedly, discontinuous AJs did not interact with previously known cortical F-actin, but, more provocatively, with prominent thick bundles of cytoplasmic actin filaments that, based on morphology and function, represent stress fibers. Moreover, the authors found that discontinuous AJs appeared to connect stress fibers between confluent endothelial cells [6], comparable to desmosomes linking intermediate filaments of, for example, adjacent epithelial cells (Figure 1). Stress fibers were known to exist in endothelial cells *in situ *[7] where they exert tension via associated non-muscle myosin II to regulate endothelial cell basal tone and contraction [8,9]. Stress fibers were also known to interact with focal adhesions (FAs), clusters of integrin-based transmembrane receptors that signal bidirectionally between intracellular and extracellular matrix milieus but, although potentially assumed in endothelia, had never before been shown to link cell-cell adhesion-mediating AJs. Discontinuous AJs attached to stress fibers did not colocalize with the FA components phospho(Y^118^)-paxillin, talin or phospho(Y^397^)-focal adhesion kinase (FAK) (Figure 1D), indicating that FAs and discontinuous AJs are distinct structures, and that discontinuous AJs can anchor the ends of stress fibers independently of FAs in confluent endothelial cells. Interestingly, discontinuous AJs do not assemble into a continuous linear morphology along the plasma membrane boundaries as is typical for AJs in epithelia, and also endothelial cells, but instead form separated clusters of VE-cadherin-based junctions at the ends of stress fibers that are oriented more or less orthogonal to the adjacent cell membranes, suggestive of tension exertion (Figure 1C). Moreover, treatment of endothelial cells with the Rho-associated coiled-coil kinase (ROCK) inhibitor Y-27632 significantly reduced the number of discontinuous AJs, although the overall level of junctional VE-cadherin remained unaltered, suggesting that discontinuous AJs are formed in response to stress fiber-induced tension acting on cell-cell junctions. Discontinuous AJs contained, aside from VE-cadherin, both α-catenin and β-catenin, as well as a number of additional junctional proteins, such as p120-catenin, plakoglobin, zona occludens (ZO)-1, junctional adhesion molecule A (JAM-A), and CD99 (Figure 1C, D), suggesting that in discontinuous AJs, VE-cadherins are physically linked to actin filament-based stress fibers in an α-catenin dependent or independent manner; possibly to stabilize these in confluent endothelial cells. Depleting vascular endothelial cells of VE-cadherin by RNA interference significantly reduced the linkage of stress fibers to cell-cell junctions, increased the number of focal adhesions, and dramatically altered the distribution and orientation of F-actin stress fibers in confluent endothelial cells.

What could explain the discrepancy between this new view of the structural organization and function of AJs compared to the earlier Nelson/Weis data described above? The latter results were obtained in E-cadherin-expressing epithelial cells (Mardin Darby canine kidney (MDCK) cells) that typically assemble into a single layered sheet of tightly bound, highly polarized cells located on the surface of organs, or the intestinal surface. These epithelia are believed to maintain a robust and permanent barrier between two contrasting environments. However, vascular endothelial cells form a polarized single-cell layer on the luminal side of blood vessels. These cellular sheets also need to establish a barrier between the blood stream and the adjacent underlying tissue to maintain vascular morphogenesis and integrity. However, endothelia and endothelial cell-cell junctions appear to be much more dynamically organized than epithelia and epithelial cell-cell junctions. Indeed, dynamic remodeling of endothelial cell monolayers in response to natural inflammatory mediators such as thrombin, endothelin and histamines, or proinflammatory stimuli such as tumor necrosis factor (TNF)-α and interleukin (IL)-1β that decrease the stability of endothelial AJs and of other cell-cell junctions (for example, GJs), are well established. These compounds promote the separation of endothelial cells to accommodate the transendothelial movement/migration (diapedesis) of leukocytes, macrophages and small solutes, or to promote endothelial cell migration in response to inflammation [10-14]. Recent work has led to the discovery of additional cell-cell adhesion modulating factors, describing α-catenin dependent and independent cell motility or adhesion-promoting homophilic E-cadherin/actin clusters [15,16], and cadherin-related proteins that, when recruited to cell-cell contacts, promote contact-dependent cell migration [17]. Thus, it is likely that different VE-cadherin-based AJ types are assembled in endothelial cells to establish either stable or transient connections to cytoskeletal F-actin filaments that in turn increase endothelial cell-cell adhesion and barrier function, or promote endothelial cell separation and migration. Data presented by Millán *et al. *[6] and others suggest that F-actin/AJ interactions in both endothelial and epithelial cells are regulated rather than constitutive. Regulated F-actin/AJ interactions would, for example, facilitate cell-cell junction remodeling during wound healing, in response to inflammatory stimuli, or to accommodate leukocyte extravasation. Future research will need to address whether and which of these AJ types require α-catenin function, and which are independent of, or promote actin-filament linkages. The concept that, in addition to the four major textbook categories of cell-cell junctions introduced above, a large number of additional cell-cell junction types with untypical sizes, shapes, and molecular compositions appear to exist that most likely serve their own special and unique functions is indeed supported by numerous structural and ultrastructural studies [18]. Thus, future research on cell-cell junctions promises to remain dynamic, and to yield unforeseeable and unanticipated results.

## About the author

MMF is a Cell Biology Professor at Lehigh University whose main research interest addresses the dynamics of gap junction biosynthesis, assembly and degradation that allows cells to modulate direct cell-to-cell communication as well as physical cell-cell coupling. He is an Associate Editor of *BMC Cell Biology*.
